# Biocompatibility, inflammatory response, and antimicrobial properties of single-bottle adhesives on gingival fibroblast and human dental pulp stem cells

**DOI:** 10.1038/s41598-026-49388-0

**Published:** 2026-04-30

**Authors:** Amir Hafez Ibrahim, Shereen Shaaban Mustafa, Nashwa El-Khazragy, Shereen Hafez Ibrahim

**Affiliations:** 1 Faculty of Dentistry, Cairo University, Cairo, Egypt; 2 Lecturer of Pediatric Dentistry and Dental Public Health Faculty of Dentistry, Beni-Suef University, Beni-Suef, Egypt; 3 Department of Clinical Pathology-Hematology, Medical Research Institute (MASRI), Faculty of Medicine, Faculty of Dentistry, Ain Shams University, Cairo, Egypt; 4 Department of Conservative Dentistry, Faculty of Dentistry, Cairo University, 11 EL-Saraya St. Manial, Cairo, 11553 Egypt

**Keywords:** Antimicrobial, Cell viability, Dental pulp stem cells, Gingival fibroblast, Universal adhesive, Cytotoxicity, Materials science, Medical research

## Abstract

The purpose of this study was to evaluate how universal adhesives affected the proliferation of gingival fibroblasts and human dental pulp stem cells by measuring cell viability at different time points: 1 min, 1 h, and 6 h. The measurement of TNF-α levels after 6 h was performed to assess any inflammatory reactions or cytokine release triggered by the materials. The antimicrobial properties against *Lactobacilli* and *Streptococcus mutans*, using the agar diffusion assay after 24 h were assessed. The single-bottle adhesives tested were: Huge Bond (Huge Dental, USA), Single Bond Universal (3 M, USA), and G-Premio BOND (GC, Japan). Huge Bond and Single Bond showed a marked decrease in cell viability after 6 h, with Single Bond exhibiting the most significant reduction (*p* < 0.0001). Huge Bond exhibited the highest TNF-α expression, followed by Single Bond and G-Premio. Huge Bond stands out for its superior performance against both bacterial strains (*p = 0.0001*). Methacrylate monomers are mostly associated with cytotoxicity if not fully polymerized because they have the ability to leach and harm dental pulp cells and gingival fibroblasts. All three materials have initial antimicrobial activity associated with the acidic monomer that was prominent with Huge Bond and G-Premio.

**Supplementary Information:**

The online version contains supplementary material available at 10.1038/s41598-026-49388-0.

## Introduction

 As part of minimally invasive dentistry, dental adhesives are developing better, multipurpose formulas that allow for strong, long-lasting bonds between restorative materials and tooth structures with additional antibacterial qualities in an effort to address the ongoing clinical problem of secondary caries and biofilm buildup at restoration margins^1^.

Dental adhesives are divided into eight generations based on how they are marketed and how many clinical procedures they require. Dental adhesive technologies have advanced since their inception in 1955, moving from etch and rinse systems in the 4th and 5th generations to self-etch systems in the 6th to 8th generations. The main objectives have been to reduce the number of bottles and processes used in dental fillings in order to shorten the procedure’s duration and strengthen the adhesion between the restoration and the tooth^2^.

As the demand for minimally invasive dental restorations and aesthetics increased, adhesive dentistry rapidly emerged. However, there is ongoing controversy over the cytotoxic effects and safe use of different dental adhesive groups. Universal adhesives are 8th generation single-bottle, multi-mode bonding agents that allow doctors to bind direct composite and some indirect ceramic restorations using self-etch, selective-etch, or total-etch procedures^3^.

Since dental adhesives come into direct or indirect touch with biological tissues, including teeth and oral soft tissues, biocompatibility is one of the most crucial properties^3^. It has been demonstrated that components of restorative resins and adhesives, including 2-hydroxyethyl methacrylate (HEMA) and triethylene glycol dimethacrylate (TEGDMA), can permeate the dentinal tubules and reach the pulp tissue in quantities that are harmful to the pulp cells^4^. In reality, following the partial polymerization of dental adhesives, a certain percentage of residual monomers is left behind^5^. The degree of conversion is the extent to which the carbon double bonds (C = C) in resin monomers are broken down into carbon single bonds (C-C) during the polymerization process of dental adhesives. Many variables influence the light-cured polymerization process, such as the light source, adhesive layer viscosity and thickness, temperature, air inhibition, solvent presence, concentrations, types and mixtures of photoinitiators, co-initiators, stabilizers, and inhibitors, as well as the types and quantities of monomers and fillers^6^.

One of the potential reasons for the negative effects of dentin adhesives was suggested to be the leakage of resin monomers^7^. Wegehaupt et al.^6^ linked the cytotoxicity of adhesives and the release of monomers to the greater degree of conversion they observed in traditional adhesives as opposed to self-etch ones. The cytotoxicity of DAs can result in allergic responses in addition to inflammation or necrosis of the oral mucosa^8^, and it would be more significant if it came into touch with crucial dentin or in situations of gingival tissue accidents^9^. As a result, the cytotoxicity of dental adhesives must be evaluated in vitro for safety evaluations.

It is debatable, nevertheless, whether some dental adhesive groups are more likely than others to cause cytotoxicity, depending on the relationship between the etching system and monomer release^10^. The fact that various studies use different methodologies and that there are several factors that might affect the results of cytotoxicity tests could add to this dispute^11–13^., and^14^. Furthermore, there is conflicting information in the literature regarding universal dental adhesives’ (UDA) potential toxicity. In dental research, their biocompatibility should be a key consideration since they are utilized in close proximity to the pulp and gingival tissues at the deeply entrenched subgingival boundary. The biocompatibility and the antimicrobial characteristics remains largely unassessed in comparison with the selected single bottle universal adhesive. No research comparing Huge Bond’s cytotoxicity to that of other universal adhesives was discovered, and studies employing dentin barrier systems indicate that etch-and-rinse adhesives are more cytotoxic. The investigation of a connection between dental adhesive type and toxicity is still hindered by the lack of methodological uniformity throughout the research. Pulp cells or gingival fibroblasts were the most used in vitro test platforms. In order to assess the antibacterial and biocompatibility of three dental materials—Huge Bond, Single Bond, and G-Premio—on human gingival fibroblast cells and dental pulp stem cells, this study was conducted. The study aimed to assess: (1) Cell Proliferation: The effect of these materials on the proliferation of human dental pulp stem cells and gingival fibroblasts by measuring cell viability at different time points: 1 min, 1 h, and 6 h. (2) Inflammatory Response: The measurement of TNF-α levels in the cells after 6 h of treatment to assess any inflammatory reactions or cytokine release triggered by the materials. (3) Antimicrobial Activity: The antimicrobial effects of the materials were evaluated against common oral pathogens, Lactobacilli and Streptococcus mutans, using the agar diffusion assay. The postulated null hypothesis was that there is no difference in cytotoxicity and antimicrobial activity between the three tested universal adhesives.

## Materials and methods

The dental adhesives used in this investigation, their descriptions, and manufacturers were presented in Table 1. The three products—Huge Bond (by Huge Dental, USA), Single Bond Universal (by 3 M, USA), and G-Premio BOND (by GC, Japan)—are dental adhesive systems used in restorative dentistry. They are designed as “universal” or “one-bottle” adhesives, meaning They can be employed in different modes; selective-etching, complete-etching, or self-etching.

**Table 1:** the materials used, specifications, composition, and manufacturer.

Universal adhesives	Specification	Composition	Manufacturer
Huge Bond	“universal” or “one-bottle” adhesives	Dimethacrylate monomer (UDMA/TEGDMA), 10-MDP (methacryloyloxydecyl dihydrogen phosphate), water, acetone, filler (silica), and initiators. It contains 10-MDP for strong, versatile bonding to various substrates (tooth, ceramic, metal).	Huge Dental, USA
Single Bond Universal		10-MDP (phosphate monomer), Bis-GMA (dimethacrylate resin), HEMA (2-hydroxyethyl methacrylate), Vitrebond copolymer (polyalkenoic acid), ethanol, water, initiators, and silane.	3 M, USA
G-Premio Bond		10-MDP, 4-MET (4-methacryloxyethyl trimellitate anhydride), MDTP (methacryloyloxydecyl dihydrogen thiophosphate), Acetone, Dimethacrylate monomers, Photoinitiators, and Silica fillers.	GC, Japan

### Study Setting

This study was conducted at Global Research Labs, Cairo, Egypt, whose facilities and technical environment greatly supported the successful completion of the work.

## Cell isolation and culture

### Isolation & culture of Human Gingival Fibroblasts (HGFs)

human gingival fibroblasts that have been separated from gingival tissue during gingivectomy or from tissue that has been discarded after dental surgery. After thoroughly rinsing the tissue with sterile phosphate-buffered saline (PBS) to get rid of any blood or debris, a sterile scalpel is used to cut it into tiny pieces (2 mm). After that, the minced tissue undergoes enzymatic digestion, frequently using a mix of dispase and collagenase (type I) enzymes. For one to two hours, the digestion process is conducted at 37 °C with mild stirring. To eliminate undigested pieces, the tissue is filtered through a sterile mesh after digestion. The fibroblasts are separated from the resulting cell suspension by centrifugation and then resuspended in α-MEM, (Catalog Number: 12571063; Gibco, Thermo Fisher), a complete culture medium supplemented with 10–20% fetal bovine serum (FBS; Gibco, Thermo Fisher, Catalog Number: 26140079); L-glutamine (Catalog Number: 25030081; Gibco, Thermo Fisher); and antibiotics (penicillin and streptomycin, Catalog Number: 15140122; Gibco, Thermo Fisher). Following seeding, the cells are cultured at 37 °C with 5% CO₂ in culture flasks. With medium changes occurring every three days, fibroblasts are permitted to multiply and cling to the culture vessels’ surface. To preserve the cells’ ability to proliferate, they are passaged and subcultured after confluence. The fibroblasts have a distinctive spindle-shaped form, and CD34 and α-smooth muscle actin (α-SMA) are used to validate their identification.

### Isolation of human dental Pulp stem cells (DPSCs)

Three wisdom teeth with clearly visible roots were used to isolate the DPSCs. material, including three fresh human dental tissue fragments. In addition to 1% DEMSO as a preservation medium, the sample was suspended in PBS (pH 7.4) containing a combination of antibiotics, including penicillin, streptomycin, and antimycotic. As soon as the sample reached the lab, the “region of interest” of the roots was carefully removed from the tooth using sterile forceps and a scalpel. The blood was then removed by washing the sample in phosphate buffered saline (PBS). The tissue was washed in PBS once more after being immersed in a 1% penicillin/streptomycin solution for a minute in order to decontaminate it from microorganisms.

The root tissue was cut into tiny pieces with forceps and sterile scissors. Afterwards, the tissue pieces were digested for an hour at 37 °C in a solution that included 0.1 U/ml of collagenase type II (Sigma-Aldrich, Germany). Collagenase (Invitrogen Life Technologies, USA) was inactivated using 1 milliliter of Dulbecco’s Modified Eagle’s Medium (Gibco, ThermoScientific) supplemented with 10% heat-inactivated foetal bovine serum (FBS) and Nutrient Mixture F-12 (DMEM/F12). For five minutes, this solution was centrifuged at 1500 rpm. After discarding the supernatant, the pellet was resuspended in 1 milliliter of DMEM/F12 with 10% FBS. T25 flasks were seeded with minced and digested dental follicle explant tissues in DMEM/F12 medium (Gibco, Thermosientific, Germany) that contained 10% foetal bovine serum (FBS) (Gibco, Thermosientific, Germany) and 1% penicillin G sodium (10.000 UI), streptomycin (10 mg), and amphotericin B (25 g) (PSA) according to Invitrogen Life Technologies. Flasks were incubated in a 5% CO2 atmosphere at 37 °C. Following the adhesion of single cells to the plastic surface, non-adherent cells were removed by switching the medium every two days. After being duplicated, plastic adherent cells achieved a confluence of around 80%. The third passage was used to conduct more experiments. With the use of CD105 and CD34, the fibroblasts’ distinctive spindle-shaped morphology is verified.

## Multiparametric characterization of DPSCs and HGFs by Flowcytometry

**Fig. 1:**
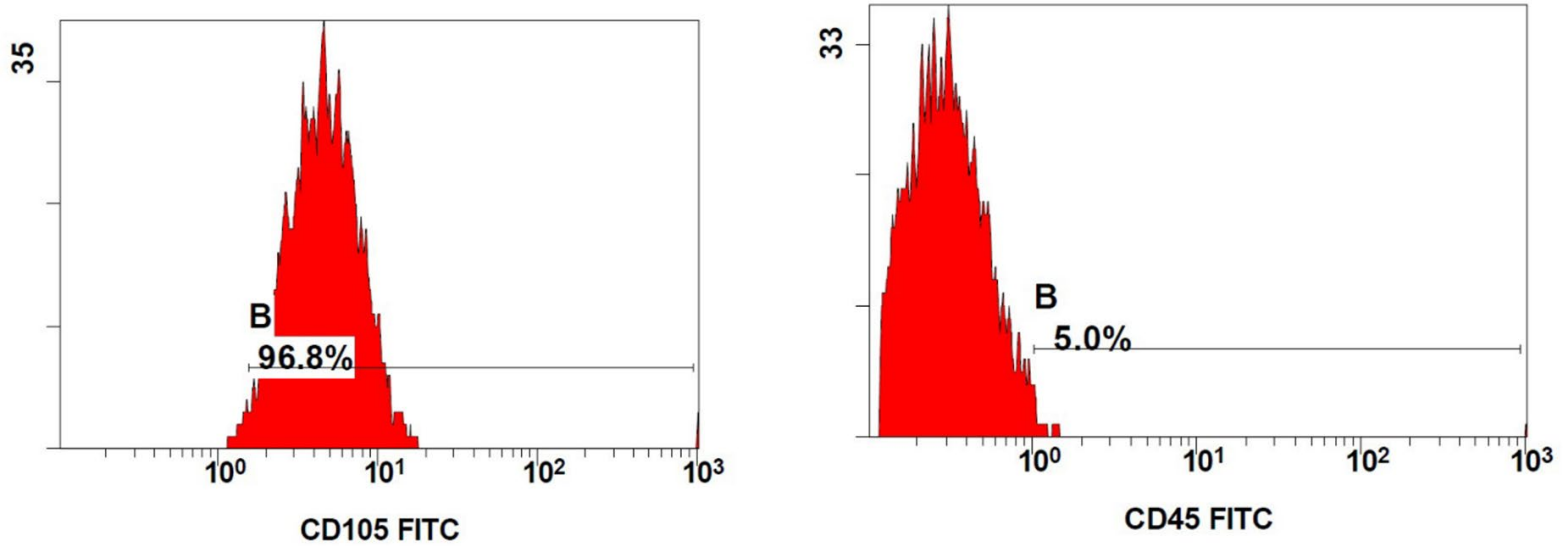
Dot-plot and fluorescence intensity histograms showed a bright expression of CD105 “MSCs specific membrane markers” in 96.8% of cells, and dim expression of CD45 “hematopoietic specific membrane markers” in 5.0% of cells, reflects pure DPSCs isolation.

**Fig. 2:**
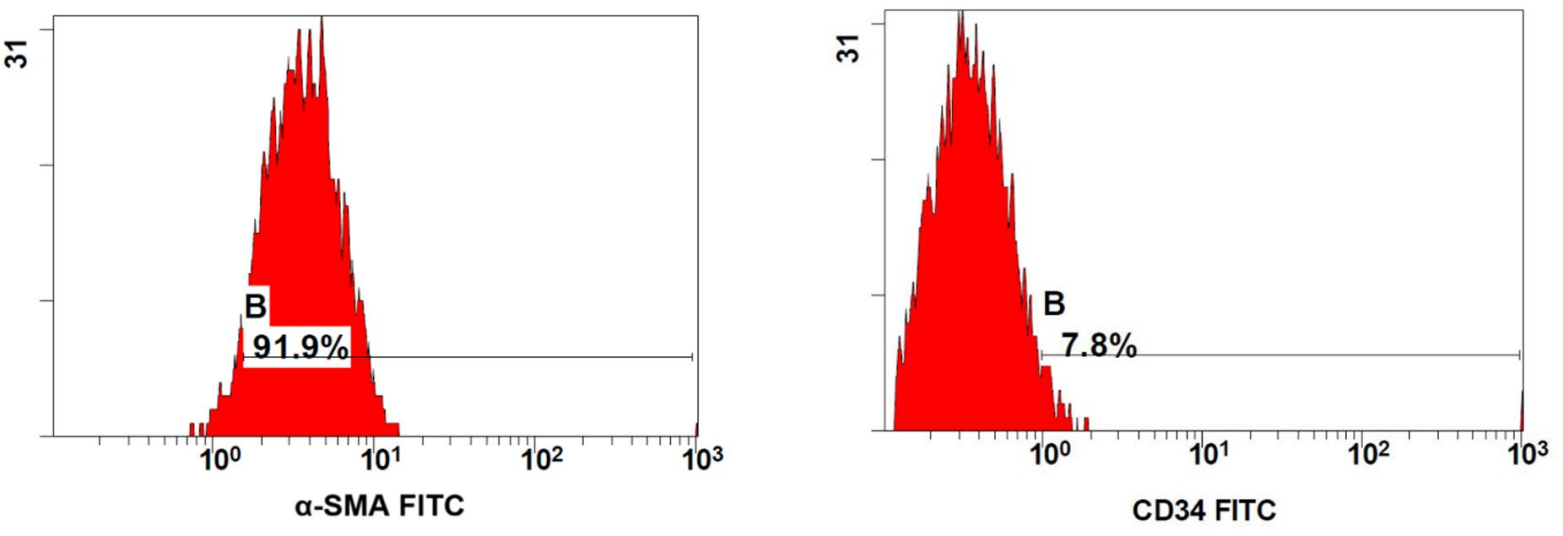
Dot-plot and fluorescence intensity histograms showed a bright expression of α-SMA “Gingival Fibroblasts specific membrane marker” in 91.9% of cells, and dim expression of CD34“hematopoietic specific membrane markers” in 7.8% of cells, reflects pure HGFs cell isolation.

**Fig. 3:**
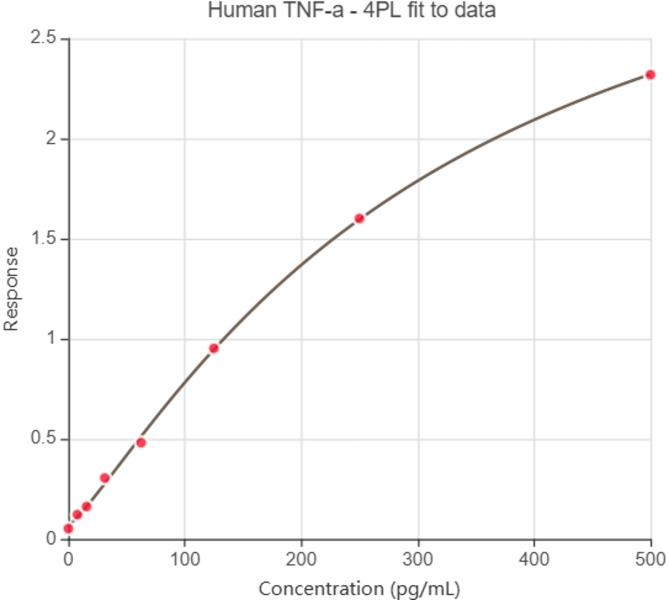
XY graph illustrating the standard curve for the Human Human TNF-α(Tumor Necrosis Factor Alpha). The X axis presents the absorbance at 492 nm, and the Y axis presents the concentrations (pg/mL).

**Fig. 4:**
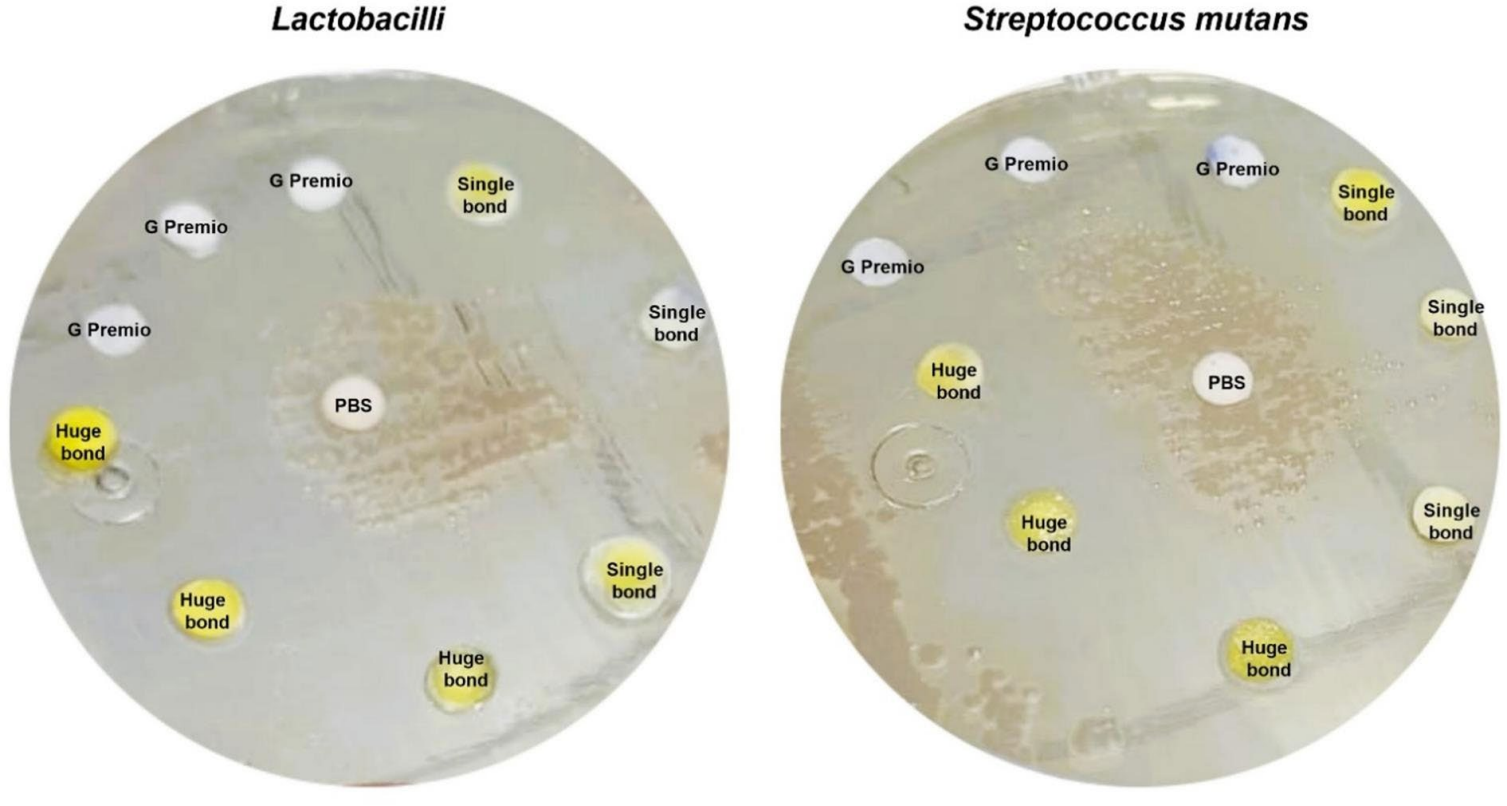
*Lactobacilli* (ATCC10241) and *Streptococcus mutants* (ATCC 25175) cultured on Brain Heart Infusion Agar (BHA) and treated with different compounds for 24 h.

The DPSC cells are stained with CD105-FITC and CD45-FITC, whereas the HGF cells are stained with α-smooth muscle actin (α-SMA) and CD34. After adjusting the count to 106/ml, the cells are suspended in PBS. The cell aspirate was disposed of after centrifugation at 800xg for 10 min, and the cell pellet was given two PBS washes. The cell pellet was immediately coated with five microliters of each monoclonal antibody. CD105 and CD45 for DPSCs and α-SMA and CD34 for HGFs are the two antibodies used separately per tube to prevent strong autofluorescence signals from arising from a larger number of cells. After 45 min of incubation at 4 °C, the cells were cleaned and reconstituted in binding buffer. Cells are gated according to their monoclonal antibody staining after the data has been processed for flowcytometric analysis. The Navios software with the NAVIOS EX 10-color flow cytometer (Beckman Coulter Life Sciences, USA): SM: To evaluate the flow cytometry data, BE14548 was utilized.(Fig. 1, 2, 3, 4)

The antibodies used to stain the DPSCs are:

*(1) CD105 Monoclonal Antibody (MEM-226)*,* FITC*,* cat no: MA1-19594*,* (Thermofisher Scientific*,* USA)*
*(2) CD45 Monoclonal Antibody (30-F11)*,* FITC*,* eBioscience*,* ThermoFisher Scientific*,* USA*.
*(3) CD34 Monoclonal Antibody*,* eBioscience*,* Cat no: PA5-85917*.
*(4) Alpha-Smooth Muscle Actin Monoclonal Antibody (1A4)*,*cat no*: ***Catalog #*** *14–9760-82 eBioscience*,* ThermoFisher Scientific*,* USA*.

The antibodies were used with a dilution of 1:100.

## Assessment of Biocompatibility Huge bond, single bond, and G Premio on of HGFs and DPSCs at three-time intervals using MTT assay

Cell viability was investigated using the MTT assay. The HGFs and DPSCs cells were prepared for the experiment using the standard trypsinization method with trypsin/EDTA one day before to the experiment. A 96-well culture plate was used for cell seeding. 200 µL of Dulbecco’s was used to seed 8 × 103 cells each well. 10% Fetal Bovine Serum (FBS) and 1% penicillin G sodium (10.000 UI), streptomycin (10 mg), and amphotericin B (25 g) were added to Modified Eagle Medium (DMEM) (Gibco, Thermosientific, Germany). To adhere cells, culture plates were incubated for 24 h at 37 °C with 5% CO2. The next day, the cells were supplemented with a steady concentration of each of the following compounds: G-Premio (GC), Huge bond, and Single bond. Additionally, control cells were treated with the carrier solvent (0.1% DMSO). For one minute, one hour, and six hours, cells were kept at 37 °C in an environment with 5% CO2. The Vybrant^®^ MTT Cell Proliferation Assay Kit, cat no. M6494 (Thermo Fisher, Germany), was used to conduct the cell cytotoxicity assay at the conclusion of the incubation period. New media was used in lieu of the 100µL of deleted media. Each well received twenty microliters of 4,5-dimethylthiazol-2-yl)−2,5-diphenyltetrazolium bromide (MTT) solution (1 mg/mL) from Invitrogen, ThermoScientific, Germany. For four hours, the plates were incubated at 37 °C with 5% CO2. Lastly, 100 µL of sodium dodecyl sulfate with hydrochloric acid (SDS-HCL) was added to each well after the MTT solution had been withdrawn. A spectrophotometer (ELx 800; Bio-Tek Instruments Inc., Winooski, VT, USA) was used to measure the optical density at 570 nm in order to assess the vitality of the cells.

## Measurement of Tumor necrosis alpha (TNF-α) in HGFs and DPSCs after treatment with Huge Bond, Single Bond, and G-Premio for 6 h by ELISA

The Enzyme-Linked Immunosorbent Assay (ELISA) was used to quantify the amount of TNF-α expressed. In accordance with the manufacturer’s instructions, the Human TNF-α (Tumor Necrosis Factor Alpha) ELISA Kit, Elabscience Biotechnology, cat no: E-EL-H0109, USA, was used to conduct the test. A microplate reader (TECAN microplate ELIZA reader, TECAN life sciences, USA) is used to measure the absorbance at 450 nm. A standard curve is used to calculate the enzyme concentration, and the results are examined appropriately.(Fig.3, 4)

## Assessment of anti-microbial effect of Huge Bond, Single Bond, and G-Premio by Agar diffusion test

Huge Bond, Single Bond, and G-Premio’s antibacterial properties against Lactobacilli and Streptococcus mutants were assessed using the Agar diffusion experiment. The bacterial strains were produced and added to the agar plates. Lactobacilli (ATCC10241) and Streptococcus mutans (ATCC 25175) are first grown in Brain Heart Infusion Agar (BHI) media for an entire night in order to create the bacterial cultures. This is followed by dilution to a standard inoculum density. Using a sterile swab, the inoculum is uniformly applied to the agar plate surface. A sterile punch is then used to make wells in the agar plates. To guarantee that the compounds are in direct contact with the agar surface, 50µL of each bonding agent, diluted 1:10 in sterile distilled water, is added to each well. To promote bacterial growth and bonding agent diffusion, the plates are incubated for 24 h at 37 °C. The antibacterial activity of the bonding agents against Lactobacilli and Streptococcus mutans is evaluated by measuring the zones of inhibition surrounding the wells following the incubation time. The efficiency of each bonding agent against bacteria is assessed by comparing the diameters of the inhibition zones to those of a conventional antibiotic or control.

### Statistical Methods

GraphPad Prism version 9 (GraphPad Software, San Diego, CA, USA) was used for all statistical analysis. Prior to analysis, the data were checked for homogeneity of variance and normality. Tukey’s post-hoc multiple comparisons test was used to ascertain pairwise significance across treatment conditions after a one-way analysis of variance (ANOVA) was used to identify overall differences for comparisons across different groups. A p-value of less than 0.05 was deemed statistically significant, and all data were presented as mean ± standard deviation. Prism’s integrated visualization capabilities were used to create the graphs, guaranteeing consistent formatting and precise depiction of all quantitative results.

## Results

### Proliferative Potential and Biocompatibility of Huge Bond, Single Bond, and G-Premio on DPSCs and HGFs: A Comparative Study

**Fig. 5:**
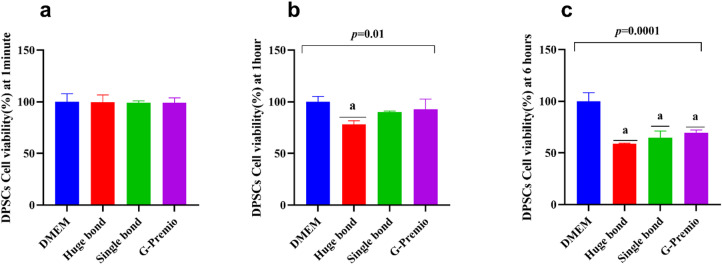
Comparative analysis of the percentage of cell viability in Dental Pulp Stem Cells (DPSCs) after exposure to different dental materials (Huge Bond, Single Bond, G-Premio) and culture media (DMEM) at three-time intervals: 1 min, 1 h, and 6 h. The data demonstrate significant differences in cell viability across the treatments, particularly at later time points.

At one-minute, one-hour, and six-hour intervals, the viability of DPSCs was evaluated when Huge Bond, Single Bond, and G-Premio were present. After six hours, the cell viability of Huge Bond and Single Bond both significantly decreased in comparison to the untreated DMEM control, with Single Bond showing the largest decline. (Fig. 5; Table 2: Cell Viability in DPSCs)

**1 min** There was no significant difference between the treatments and DMEM, indicating that all materials have a comparable short-term effect on cell viability.

**1 h** At this point, Huge Bond exhibited a noticeable decline in viability (78.1%), while Single Bond and G-Premio were relatively better maintained (89.9% and 92.9%, respectively). The difference between Huge Bond and DMEM was statistically significant (*p* = 0.01), indicating that Huge Bond has a mild adverse effect after 1 h of exposure.

**6 h** The most significant effects were observed after 6 h of treatment. Huge Bond (59%) and Single Bond (64.7%) demonstrated significantly reduced cell viability compared to DMEM (100%). In contrast, G-Premio (69.3%) showed better cell viability but still had a significant difference compared to DMEM (*p* < 0.0001). These results suggest that both Huge Bond and Single Bond have a considerable negative impact on DPSCs over time, while G-Premio shows a relatively higher biocompatibility.

**Table 2:** Descriptive statistics for the percentage of cell viability in DPSCs after culture in different media at three-time intervals.

Group	mean ± SD			*P*-value
	1 min	1 h	6 h	
DMEM	100 ± 7.76	100 ± 5.18	100 ± 8.36	> 0.05
Huge bond	99.8 ± 6.85	78.1 ± 3.6^a^	59.0 ± 0.48^a^	0.01
Single bond	99.3 ± 1.63	89.9 ± 1.19	64.7 ± 6.41^a^	0.02
G Premio	98.9 ± 4.95	92.9 ± 9.88	69.3 ± 2.91^a^	0.02
*p*-value	0.99	0.01	< 0.0001	

ANOVA: Analysis of variance test. statistical significance compared to the DPSCs “untreated cells” (*p* < 0.05), DPSCs: Dental pulp- derived mesenchymal stem cells, DMEM: Dulbecco’s Modified Eagle Medium (DMEM).^a^: Statistical significance compared to cells cultured in DMEM.

**Fig. 6:**
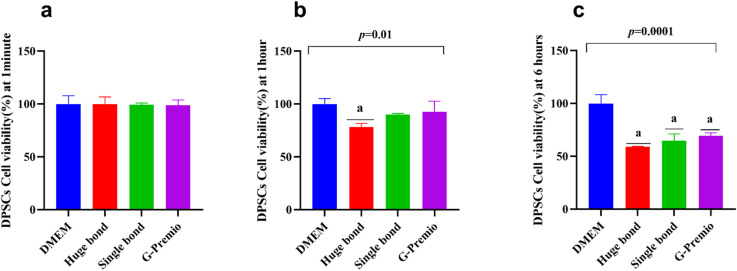
Comparative analysis of the percentage of cell viability in Human Gingival Fibroblasts (HGFs) exposed to Huge Bond, Single Bond, G-Premio, and culture media (DMEM) at 1 min, 1 h, and 6 h. Statistical differences are observed in all groups, showing variation in biocompatibility across the materials.

The viability of HGFs was similarly tested at 1 min, 1 h, and 6 h. Huge Bond and Single Bond were both found to significantly reduce cell viability across all time intervals, with Huge Bond exhibiting the greatest reduction. (Table 3; Fig. 6: Cell Viability in HGFs)

**1 min** Huge Bond showed a slight reduction in cell viability (94.5%) compared to the DMEM control, but this was not statistically significant. The other materials, including Single Bond and G-Premio, showed near-normal cell viability.

**1 h** After 1-hour, Huge Bond (72.0%) exhibited a significant reduction in viability compared to DMEM (100%), with Single Bond (82.9%) and G-Premio (87.5%) showing a lesser decrease. The statistical significance (*p* = 0.001) suggests that Huge Bond has a more adverse effect on HGFs at this time.

**6 h** After 6 h, Huge Bond demonstrated the most significant decrease in cell viability (51.7%), followed by Single Bond (57.6%) and G-Premio (65.5%). All these reductions were statistically significant when compared to DMEM. This further reinforces that Huge Bond may not be as biocompatible for HGFs as the other materials.

**Table 3:** Descriptive statistics for the percentage of cell viability in HGFs after culture in different media at three-time intervals.

Group	mean ± SD			*P*-value
	1 min	1 h	6 h	
DMEM	100 ± 4.94	100 ± 7.90	100 ± 6.10	> 0.05
Huge bond	94.5 ± 11.1	72.0 ± 3.52^a/d^	51.7 ± 0.54^a/d^	0.02
Single bond	92.6 ± 9.61	82.9 ± 2.73^a^	57.6 ± 6.99 ^a^	0.03
G Premio	97.0 ± 3.43	87.5 ± 2.70^a/b^	65.5 ± 1.75 ^a/b^	0.01
*p*-value	0.699	0.0007	0.0001	

ANOVA: Analysis of variance test. statistical significance compared to the DPSCs “untreated cells” (*p* < 0.05), DPSCs: Dental pulp- derived mesenchymal stem cells, DMEM: Dulbecco’s Modified Eagle Medium (DMEM). ^a^: Statistical significance compared to cells cultured in DMEM, ^b^: Statistical significance compared to cells cultured in Huge bond, ^c^: Statistical significance compared to cells cultured in Single bond, ^d^: Statistical significance compared to cells cultured in G. Premio.

## TNF-α Expression and Antimicrobial Activity of Huge Bond, Single Bond, and G-Premio

### TNF-α Expression in DPSCs (Table 4; Fig. 7a)

The TNF-α levels in DPSCs after exposure to Huge Bond, Single Bond, and G-Premio show significant variation. Huge Bond exhibited the highest TNF-α expression, followed by Single Bond and G-Premio, which both showed a comparatively lower increase.

At 6 h, Huge Bond caused a substantial increase in TNF-α expression (17.6 ± 0.72), which was statistically significant compared to DMEM control (2.25 ± 0.23) as indicated by the a, c, and d markers. Single Bond also significantly elevated TNF-α expression (13.5 ± 0.73), while G-Premio (10.5 ± 1.22) had the lowest expression among the tested materials. These findings suggest that Huge Bond and Single Bond trigger a more significant inflammatory response in DPSCs compared to G-Premio, which seems to be less inflammatory.

**Table 4:** Descriptive statistics for the TNF-α expression of three compounds in DPSCs & HGFs after culture in different media for 6 h.

Group	mean ± SD		*P*-value
	DPSCs	HGFs	
DMEM	2.25 ± 0.228	2.61 ± 0.34	> 0.05
Huge bond	17.6 ± 0.72^a/c/d^	18.9 ± 1.34 ^a/c/d^	> 0.05
Single bond	13.5 ± 0.73 ^a/b/d^	13.2 ± 1.25 ^a/b/d^	> 0.05
G Premio	10.5 ± 1.22 ^a/b/c^	8.78 ± 1.42 ^a/b/c^	> 0.05
*p*-value	0.0001	0.0001	

ANOVA: Analysis of variance test. statistical significance compared to the DPSCs “untreated cells” (*p* < 0.05), DPSCs: Dental pulp- derived mesenchymal stem cells, DMEM: Dulbecco’s Modified Eagle Medium (DMEM). ^a^: Statistical significance compared to cells cultured in DMEM, ^b^: Statistical significance compared to cells cultured in Huge bond, ^c^: Statistical significance compared to cells cultured in Single bond, ^d^: Statistical significance compared to cells cultured in G. Premio.

### TNF-α Expression in HGFs (Table 4; Fig. 7b)

Similar to DPSCs, Huge Bond caused the highest TNF-α expression in HGFs, followed by Single Bond and G-Premio. After 6 h of treatment, Huge Bond induced the highest TNF-α production (18.9 ± 1.34), which was significantly different from the control (2.61 ± 0.34). Single Bond and G-Premio also caused significant increases in TNF-α levels, although the levels were lower than Huge Bond. These results further support that Huge Bond has a higher inflammatory potential, affecting both DPSCs and HGFs.

**Fig. 7:**
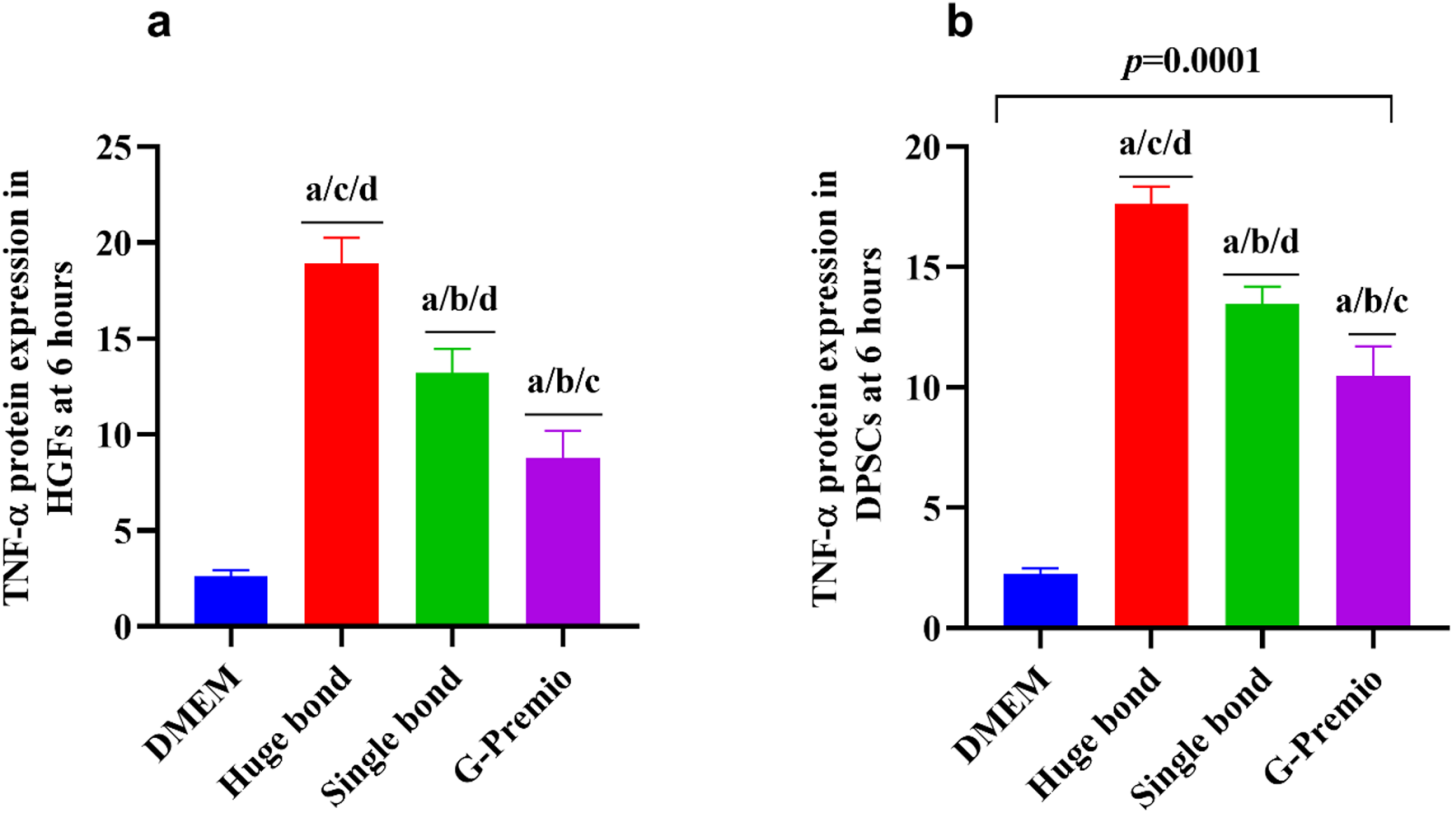
TNF-α Expression in DPSCs and HGFs after treatment with Huge Bond, Single Bond, and G-Premio for 6 h. The bar graphs show the protein expression levels of TNF-α in both DPSCs (Dental Pulp Stem Cells) and HGFs (Human Gingival Fibroblasts). Significant differences are indicated by the statistical markers between treatments and DMEM control.

## Antimicrobial Activity of Huge Bond, Single Bond, and G-Premio

### Inhibition Zone on *Lactobacilli* (Fig. 8a)

The antimicrobial activity of Huge Bond, Single Bond, and G-Premio was evaluated by measuring the inhibition zones around each material against *Lactobacilli*. Huge Bond produced the largest inhibition zone (5.93 ± 0.51 mm) compared to the other materials, followed by G-Premio (5.50 ± 0.44 mm) and Single Bond (4.47 ± 0.73 mm). The statistical significance (*p = 0.0001*) indicates that Huge Bond is significantly more effective at inhibiting *Lactobacilli* growth than Single Bond and G-Premio, though the latter two showed moderate inhibition.

### Inhibition Zone on *Streptococcus mutans* (Table 5; Fig. 8b)

The antimicrobial effects were also tested against *Streptococcus mutans*, a key bacterium in dental caries formation. Huge Bond again showed the largest inhibition zone (5.50 ± 0.88 mm), significantly larger than both Single Bond (4.23 ± 0.25 mm) and G-Premio (4.93 ± 0.23 mm). The statistical analysis (*p = 0.0001*) indicated that Huge Bond was significantly more effective than Single Bond and G-Premio in inhibiting *Streptococcus mutans*. While all three materials demonstrated some antimicrobial activity, Huge Bond stands out for its superior performance against both bacterial strains.

**Table 5:** Descriptive statistics for the Zone inhibition (%) of three materials on *Lactobacilli* and *Streptococcus mutants* after culture for 48 h.

Group	mean ± SD		*P*-value
	Lactobacilli	S. mutans	
PBS	0.0	0	
Huge bond	5.93 ± 0.51^a/c^	5.50 ± 0.88 ^a/c^	> 0.05
Single bond	4.47 ± 0.73^a/b^	4.23 ± 0.25 ^a/b^	> 0.05
G Premio	5.50 ± 0.44^a^	4.93 ± 0.23 ^a^	> 0.05
*p*-value	0.0001	0.0001	> 0.05

ANOVA: Analysis of variance test. statistical significance compared to the DPSCs “untreated cells” (*p* < 0.05), DPSCs: Dental pulp- derived mesenchymal stem cells, DMEM: Dulbecco’s Modified Eagle Medium (DMEM). ^a^: Statistical significance compared to cells cultured in DMEM, ^b^: Statistical significance compared to cells cultured in Huge bond, ^c^: Statistical significance compared to cells cultured in Single bond, ^d^: Statistical significance compared to cells cultured in G. Premio.

**Fig. 8:**
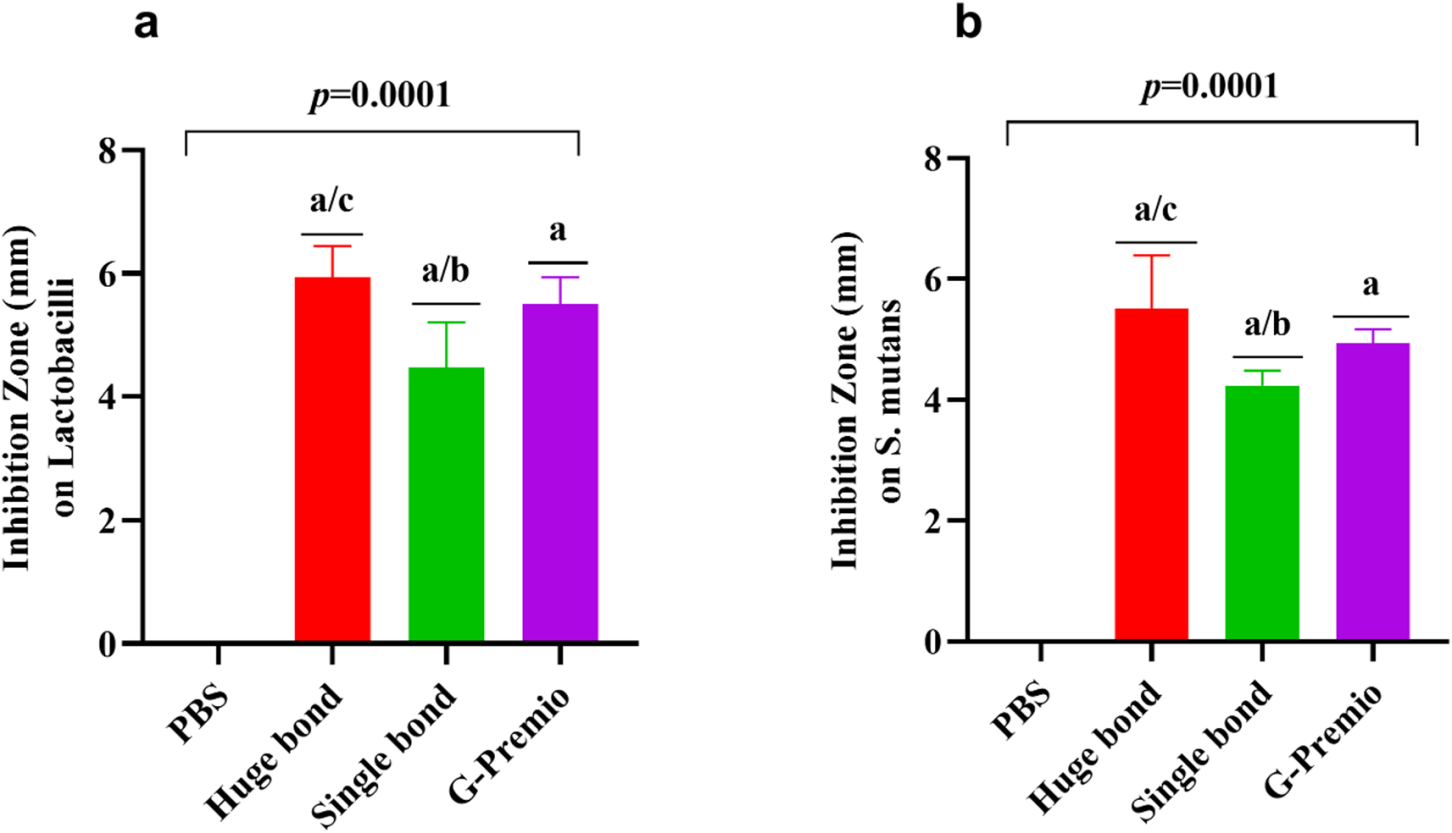
*Inhibition Zones of Huge Bond*,* Single Bond*,* and G-Premio* against *Lactobacilli* and *Streptococcus mutans* after 48 h of culture. The graph compares the inhibition zones formed around each material, demonstrating their antimicrobial effectiveness.

## Discussion

Research in dentistry should prioritize the biocompatibility of dental adhesives since they are utilized in close proximity to the pulp and to gingival tissues in deeply seated subgingival margin In vitro study of a material’s cytocompatibility is crucial for determining its potential for clinical use.

The purpose of this study was to evaluate the biocompatibility of three dental materials “Huge Bond, Single Bond, and G-Premio” on human dental stem cells and human gingival fibroblast cells. Moreover, their antimicrobial properties against S. Mutans and Lactobacilli were assessed. Based on the findings of this study, the null hypothesis tested was rejected.

“Cytotoxicity” should be interpreted as a broader biological spectrum—including not only short-term viability but also inflammatory signaling and pro-regenerative differentiation capacity under eluate- and dose-dependent conditions^15^. The ISO 10993-5 standard, issued by the International Organization for Standardisation in 2009, offers thorough criteria for assessing the cytotoxicity of materials used in Medical uses^15^. This study followed ISO 10993-5 guidelines and utilised the MTT test to assess the cytotoxicity of dental materials. The MTT test is widely recognized as a standard tool for evaluating the cytotoxicity of biomaterials, especially in the dentistry industry.

The materials selected in the current study are dental adhesives used in restorative dentistry. They are designed as “universal” or “one-bottle” adhesives, meaning they can be used in multimodes. However, their chemical compositions differ significantly. They are mixtures, rather than single molecules, designed to be hydrophilic enough to wet tooth structure and hydrophobic enough to prevent long-term degradation. HugeBond and G-Premio are modern universal adhesives containing functional monomers, while “Single Bond” refers to a basic covalent bond in chemistry. As claimed by the manufacturers, Huge Bond is described as an 8th-generation universal adhesive. The key Components were Dimethacrylate monomer (often UDMA/TEGDMA), 10-MDP for chemical bonding to tooth and metals, water, acetone, filler (silica), and initiators. It contains 10-MDP for strong, versatile bonding to various substrates (tooth, ceramic, metal). Single Bond Universal is a 7th/8th generation adhesive. It is composed of 10-MDP, Bis-GMA, HEMA, Vitrebond copolymer (polyalkenoic acid), ethanol, water, initiators, and silane. It contains 10-MDP for bonding, HEMA for improving infiltration, and a silane component allowing it to bond directly to silica-based ceramics. G-Premio BOND is a 7th/8th generation one-bottle universal adhesive. It is composed of 10-MDP, 4-MET, MDTP, Acetone, Dimethacrylate monomers, Photoinitiators, and Silica fillers. It is a HEMA-free formulation, designed to reduce water absorption over time. It is highly versatile due to a combination of three functional monomers (MDP, 4-MET, MDTP). The selection of the three adhesives was mainly based on the assessment of the difference in the active functional monomers: 10-MDP (in all three), 4-MET (in G-Premio), MDTP (in G-Premio). Meanwhile, these bond to hydroxyapatite and metal oxides. The difference in the polymerizable resins: Dimethacrylates (UDMA, Bis-GMA), HEMA (in Single Bond), TEGDMA and the solvents whether Acetone (G-Premio, Huge Bond), Ethanol (Single Bond), Water. All the tested compounds have Silica filler (SiO2) and their pH Level are acidic, typically with a pH around 1.5 to 2.7, allowing them to etch the tooth surface.

The chemical composition of Huge Bond, Single Bond, and G-Premio directly influences their cytotoxicity and antimicrobial properties because the types of monomers, solvents, and additives determine how toxic they are to oral cells and whether they inhibit bacterial growth. Dental materials containing cytotoxic components can impair cell integrity, causing pyknotic nuclei and cytoskeletal structure changes^15^. Despite the possibility of such deleterious consequences and based on the findings of the present study, the materials evaluated in this investigation did not show cytological alterations indicative of cytotoxic reactions when immediately tested. However, at one and six hours, severe cytotoxic effects were observed with different tested materials.

The results of the MTT and migration studies revealed the absence of cytological damage upon immediate application of the adhesives, indicating that the tested materials are biocompatible under the given conditions.

The role of oxidative stress in this context is critical, as materials that induce higher levels of reactive oxygen species (ROS) are often associated with increased cytotoxicity and genotoxicity. Excessive ROS production can DNA damaging effect, shorten telomeres, and accelerate cell senescence, resulting in cell death^15,16^. The results from Tables 2, 3, 4 and 5 indicate that the biocompatibility of Huge Bond, Single Bond, and G-Premio varies considerably over time and between cell types. All materials were biocompatible when immediately placed (one minute cell contact). Huge Bond has a mild adverse effect after 1 h of exposure. Huge Bond and Single Bond appear to have the most negative effect on both DPSCs and HGFs, particularly over extended exposure times (6 h). Single Bond also significantly reduces cell viability, especially in DPSCs, though its impact on HGFs is less severe. G-Premio, on the other hand, demonstrates the least negative impact, particularly for DPSCs. This might be attributed to that G-Premio doesn’t contain methacrylate functional monomer. However, it still shows a reduction in cell viability compared to the control. This dictates the importance to ensure adequate and complete photopolymerization of the adhesives to avoid the presence of any residual monomer that could elicit cytotoxic effect on the pupal tissues in deep cavities or on the gingival tissues in deep cervical or proximal cavity margins. Exposing cells to cytotoxic components in dental materials can cause structural alterations, including nuclear pyknosis and cytoskeletal disorganization^17^.

The cytotoxicity of huge bond might be attributed to Bis-GMA and HEMA that can leach out before complete polymerization, leading to cytotoxic effects on pulp cells and fibroblasts. The presence of MDP improves bonding but does not reduce cytotoxicity significantly. Although, Huge Bond does not contain specific antimicrobial agents, its antimicrobial effect might be attributed to low pH during etching, which can inhibit bacterial growth. As revealed in this study, the statistical significance (*p = 0.0001*) indicates that Huge Bond is significantly more effective at inhibiting *Lactobacilli* and *Streptococcus mutans* growth than Single Bond and G-Premio, though the latter two showed moderate inhibition. While all three materials demonstrated some antimicrobial activity, Huge Bond stands out for its superior performance against both bacterial strains.

The current study’s findings demonstrated consistency across all tests and were consistent with the findings that were previously obtained from other studies, including Adhese Universal (Ivoclar Vivadent, Schaan, Liechtenstein); OptiBond Universal (Kerr, Brea, CA, USA); and Prime & Bond Universal (Dentsply Sirona, Charlotte, NC, USA)^15,18^. These variations in cytotoxicity may be caused by variations in the particular adhesive’s composition, specifically in the ratio of the compounds and the distinct monomers. It was mentioned that the composition of dental adhesives may contain varying amounts of methacrylate monomers, including TEGDMA, UDMA, HEMA, PENTA, and bis-GMA, which might affect how harmful they are. When compared to the individual monomers, their combined hazardous impact may also be amplified due to their synergistic interactions^19^. According to the literature, bis-GMA had the highest cytotoxicity among the methacrylate monomers, whereas UDMA, HEMA, and TEGDMA demonstrated the lowest cytotoxicity^15–22^. Bis-GMA has a comparatively high level of cytotoxicity among the monomers used in dental adhesives. Due to its large molecular weight, this monomer has a limited capacity to pass through dentin; yet, it is prone to hydrolysis, and its byproducts may cause a reduction in the permeability of cell membranes^22^.

Furthermore, it was reported that intracellular glutathione (GSH) was depleted and apoptosis was induced in human gingival fibroblasts treated with bis-GMA. GSH depletion, cell cycle arrest, apoptosis/necrosis induction, and ROS generation are the mechanisms by which UDMA toxicity is brought on. Additionally, it increases the mRNA expression of cyclo-oxygenase-2, heme oxygenase-1, and carboxylesterase-2 in pulp cells^16^.

Regarding the cytotoxicity of Single Bond, it was attributed to the high HEMA content that increases cytotoxicity because HEMA diffuses easily into dentin and pulp tissues. The residual monomers can cause oxidative stress and apoptosis in pulp cells. While it has minimal antimicrobial activity. Studies show that HEMA can cause toxicity in the cells that make up pulp tissue and has the ability to diffuse quickly into dentin. According to certain reports, it can cause morphological alterations in a variety of cell types as well as apoptosis and growth retardation^23–25^.

Regarding G-Premio Bond, studies show moderate cytotoxicity similar to other universal adhesives, mainly due to unpolymerized monomers. The combination of multiple functional monomers does not eliminate toxicity but enhances bonding efficiency. While G-Premio has slight antibacterial effects due to its acidic monomers (MDP, 4-MET), which lower pH and inhibit bacterial metabolism temporarily. However, it lacks sustained antimicrobial activity unless modified with antibacterial additives. It was reported that 10-MDP demonstrated an inflammatory response and inhibited odontoblastic development of human pulp cells^26^. By interacting directly with cells that resemble odontoblasts, it can also cause mineralization depression^27^. In contrast, Nakagawa et al. claim that testing 4-MET in resin-containing luting materials revealed biocompatibility in dental pulp cells^28,29^. Furthermore, it was shown that the dental adhesives may become more hazardous as a result of lower pH values^30^. It was reported that for a minimum of twenty-four hours, one-bottle bonding agents exhibit bactericidal qualities in vitro^31^. Thus, “cytotoxicity” should be interpreted as a broader biological spectrum—including not only short-term viability but also inflammatory signaling and pro-regenerative differentiation capacity under eluate- and dose-dependent conditions^17^. Moreover, the degree of polymerization and the presence of unreacted monomer should be considered.

Our anti-bacterial findings were in accordance with other studies^32–34^ where it was reported that the elution of certain unpolymerized components present in the adhesive system, which are often poisonous to the bacterial colony, or the acidity of the functional monomer 10-MDP may be responsible for the antibacterial action^33^. Furthermore, agents with anti-biofilm properties served as an excellent inhibitor of cariogenic virulence, suppressed the growth of S. mutans, and compromise the acidogenicity^34^.

This study might suffer from certain constraints. The tests’ in vitro nature is the main source of the current study’s shortcomings. We obtained similar results in a variety of tests based on different molecular mechanisms of cytotoxicity, genotoxicity, inflammatory response and antimicrobial assessment, indicating the high credibility of the methods used. The results were comparable to other studies^18^ based on different adhesives using a similar methodology. Furthermore, due to the absence of a dentin barrier, smear layer, and immune response, the in-vitro results are difficult to relate to a clinical scenario that would occur in a tooth cavity. Moreover, in the present studies the adhesives tested were not polymerized therefore more studies is required to test the cytotoxic reaction and the antimicrobial properties of these adhesives when freshly polymerized and for long time follow up.

## Conclusions

Within the constraints of the present research, the following conclusions were derived: The effects of each tested universal adhesive on gingival fibroblasts and dental pulp stem cells vary according to the chemical formulation. Every tested adhesive was biocompatible when applied in the first minute before curing. Methacrylate monomers (Bis-GMA, HEMA, and TEGDMA) are mostly associated with cytotoxicity if not fully polymerized because they have the ability to leach and harm dental pulp cells and gingival fibroblasts. The antimicrobial activity of the tested universal adhesives is associated to the acidic monomer that was more prominent with HugeBond and G-Premio.

## Electronic Supplementary Material

Below is the link to the electronic supplementary material.

Supplementary Material 1

## Data Availability

All data generated are included in the current manuscript and available upon reasonable request to the corresponding author.
