# Supplementary material for: Biocompatibility, inflammatory response, and antimicrobial properties of single-bottle adhesives on gingival fibroblast and human dental pulp stem cells
Source: Sci Rep. 2026 Apr 30;16:13886. doi: 10.1038/s41598-026-49388-0 (PMC13133283; doi:10.1038/s41598-026-49388-0)
Supplement: Supplementary file 1 — Supplementary Material 1 [file 41598_2026_49388_MOESM1_ESM.docx]

**Supplementary tables**

**Table S1. Tukey's multiple comparisons test for the percentage of cell viability after treatment of DPSCs for 1 minute with the tested materials**

| **Tukey's multiple comparisons test** | **Mean Diff.** | **95.00% CI of diff.** | **Adjusted P Value** |
| --- | --- | --- | --- |
| DMEM vs. Huge bond | 0.2100 | -14.94 to 15.36 | >0.9999 |
| DMEM vs. Single bond | 0.6633 | -14.49 to 15.82 | 0.9989 |
| DMEM vs. G-Premio | 1.097 | -14.06 to 16.25 | 0.9953 |
| Huge bond vs. Single bond | 0.4533 | -14.70 to 15.61 | 0.9997 |
| Huge bond vs. G-Premio | 0.8867 | -14.27 to 16.04 | 0.9975 |
| Single bond vs. G-Premio | 0.4333 | -14.72 to 15.59 | 0.9997 |

CI: Confidence interval, DPSCs: Dental pulp- derived mesenchymal stem cells**,** DMEM: Dulbecco's Modified Eagle Medium (DMEM)**.**

**Table S2. Tukey's multiple comparisons test for the percentage of cell viability after treatment of DPSCs for 1 hour with the tested materials**

| **Tukey's multiple comparisons test** | **Mean Diff.** | **95.00% CI of diff.** | **Adjusted P Value** |
| --- | --- | --- | --- |
| DMEM vs. Huge bond | 21.94 | 6.544 to 37.34 | 0.0080 |
| DMEM vs. Single bond | 10.08 | -5.316 to 25.48 | 0.2327 |
| DMEM vs. G-Premio | 7.137 | -8.259 to 22.53 | 0.4881 |
| Huge bond vs. Single bond | -11.86 | -27.26 to 3.536 | 0.1407 |
| Huge bond vs. G-Premio | -14.80 | -30.20 to 0.5923 | 0.0595 |
| Single bond vs. G-Premio | -2.943 | -18.34 to 12.45 | 0.9253 |

CI: Confidence interval, DPSCs: Dental pulp- derived mesenchymal stem cells**,** DMEM: Dulbecco's Modified Eagle Medium (DMEM)**.**

**Table S3. Tukey's multiple comparisons test for the percentage of cell viability after treatment of DPSCs for 6 hours with the tested materials**

| **Tukey's multiple comparisons test** | **Mean Diff.** | **95.00% CI of diff.** | **Adjusted P Value** |
| --- | --- | --- | --- |
| DMEM vs. Huge bond | 41.03 | 26.73 to 55.33 | <0.0001 |
| DMEM vs. Single bond | 35.27 | 20.97 to 49.57 | 0.0002 |
| DMEM vs. G-Premio | 30.67 | 16.37 to 44.97 | 0.0006 |
| Huge bond vs. Single bond | -5.757 | -20.06 to 8.543 | 0.5937 |
| Huge bond vs. G-Premio | -10.36 | -24.66 to 3.943 | 0.1725 |
| Single bond vs. G-Premio | -4.600 | -18.90 to 9.700 | 0.7377 |

CI: Confidence interval, DPSCs: Dental pulp- derived mesenchymal stem cells**,** DMEM: Dulbecco's Modified Eagle Medium (DMEM)**.**

**Table S4. Tukey's multiple comparisons test for the percentage of cell viability after treatment of HGFs for 1 minute with the tested materials**

| **Tukey's multiple comparisons test** | **Mean Diff.** | **95.00% CI of diff.** | **Adjusted P Value** |
| --- | --- | --- | --- |
| DMEM vs. Huge bond | 5.500 | -15.22 to 26.22 | 0.8296 |
| DMEM vs. Single bond | 7.403 | -13.31 to 28.12 | 0.6748 |
| DMEM vs. G-Premio | 3.043 | -17.67 to 23.76 | 0.9635 |
| Huge bond vs. Single bond | 1.903 | -18.81 to 22.62 | 0.9905 |
| Huge bond vs. G-Premio | -2.457 | -23.17 to 18.26 | 0.9801 |
| Single bond vs. G-Premio | -4.360 | -25.08 to 16.36 | 0.9041 |

CI: Confidence interval, DPSCs: Dental pulp- derived mesenchymal stem cells**,** DMEM: Dulbecco's Modified Eagle Medium (DMEM)**.**

**Table S5. Tukey's multiple comparisons test for the percentage of cell viability after treatment of HGFs for 1 hour with the tested materials**

| **Tukey's multiple comparisons test** | **Mean Diff.** | **95.00% CI of diff.** | **Adjusted P Value** |
| --- | --- | --- | --- |
| DMEM vs. Huge bond | 27.94 | 15.57 to 40.31 | 0.0004 |
| DMEM vs. Single bond | 17.08 | 4.714 to 29.45 | 0.0095 |
| DMEM vs. G-Premio | 12.46 | 0.09355 to 24.83 | 0.0483 |
| Huge bond vs. Single bond | -10.85 | -23.22 to 1.516 | 0.0870 |
| Huge bond vs. G-Premio | -15.47 | -27.84 to -3.104 | 0.0165 |
| Single bond vs. G-Premio | -4.620 | -16.99 to 7.750 | 0.6459 |

CI: Confidence interval, DPSCs: Dental pulp- derived mesenchymal stem cells**,** DMEM: Dulbecco's Modified Eagle Medium (DMEM)**.**

**Table S6. Tukey's multiple comparisons test for the percentage of cell viability after treatment of HGFs for 6 hours with the tested materials**

| **Tukey's multiple comparisons test** | **Mean Diff.** | **95.00% CI of diff.** | **Adjusted P Value** |
| --- | --- | --- | --- |
| DMEM vs. Huge bond | 48.27 | 35.91 to 60.62 | <0.0001 |
| DMEM vs. Single bond | 42.39 | 30.03 to 54.75 | <0.0001 |
| DMEM vs. G-Premio | 34.44 | 22.08 to 46.80 | <0.0001 |
| Huge bond vs. Single bond | -5.877 | -18.23 to 6.480 | 0.4683 |
| Huge bond vs. G-Premio | -13.83 | -26.18 to -1.470 | 0.0294 |
| Single bond vs. G-Premio | -7.950 | -20.31 to 4.407 | 0.2441 |

CI: Confidence interval, DPSCs: Dental pulp- derived mesenchymal stem cells**,** DMEM: Dulbecco's Modified Eagle Medium (DMEM)**.**

**Table S7. Tukey's multiple comparisons test for the TNF-α after treatment of DPSCs for 6 hours with the tested materials**

| **Tukey's multiple comparisons test** | **Mean Diff.** | **95.00% CI of diff.** | **Adjusted P Value** |
| --- | --- | --- | --- |
| DMEM vs. Huge bond | -15.37 | -17.48 to -13.26 | <0.0001 |
| DMEM vs. Single bond | -11.22 | -13.33 to -9.105 | <0.0001 |
| DMEM vs. G-Premio | -8.233 | -10.34 to -6.121 | <0.0001 |
| Huge bond vs. Single bond | 4.156 | 2.045 to 6.268 | 0.0010 |
| Huge bond vs. G-Premio | 7.140 | 5.028 to 9.252 | <0.0001 |
| Single bond vs. G-Premio | 2.984 | 0.8721 to 5.095 | 0.0084 |

CI: Confidence interval, DPSCs: Dental pulp- derived mesenchymal stem cells**,** DMEM: Dulbecco's Modified Eagle Medium (DMEM)**.**

**Table S8. Tukey's multiple comparisons test for the TNF-α after treatment of HSFs for 6 hours with the tested materials**

| **Tukey's multiple comparisons test** | **Mean Diff.** | **95.00% CI of diff.** | **Adjusted P Value** |
| --- | --- | --- | --- |
| DMEM vs. Huge bond | -16.32 | -19.38 to -13.26 | <0.0001 |
| DMEM vs. Single bond | -10.61 | -13.67 to -7.546 | <0.0001 |
| DMEM vs. G-Premio | -6.172 | -9.235 to -3.110 | 0.0009 |
| Huge bond vs. Single bond | 5.710 | 2.648 to 8.773 | 0.0015 |
| Huge bond vs. G-Premio | 10.15 | 7.084 to 13.21 | <0.0001 |
| Single bond vs. G-Premio | 4.436 | 1.374 to 7.499 | 0.0072 |

CI: Confidence interval, DPSCs: Dental pulp- derived mesenchymal stem cells**,** DMEM: Dulbecco's Modified Eagle Medium (DMEM)**.**

**Table S9. Tukey's multiple comparisons test for the inhibition zone (mm) of Lactobacilli after treatment for 48 hours with the tested materials**

| **Tukey's multiple comparisons test** | **Mean Diff.** | **95.00% CI of diff.** | **Adjusted P Value** |
| --- | --- | --- | --- |
| PBS vs. Huge bond | -5.933 | -7.239 to -4.628 | <0.0001 |
| PBS vs. Single bond | -4.467 | -5.772 to -3.161 | <0.0001 |
| DPBS vs. G-Premio | -5.500 | -6.805 to -4.195 | <0.0001 |
| Huge bond vs. Single bond | 1.467 | 0.1615 to 2.772 | 0.0288 |
| Huge bond vs. G-Premio | 0.4333 | -0.8718 to 1.739 | 0.7197 |
| Single bond vs. G-Premio | -1.033 | -2.339 to 0.2718 | 0.1280 |

CI: Confidence interval, DPSCs: Dental pulp- derived mesenchymal stem cells**,** DMEM: Dulbecco's Modified Eagle Medium (DMEM)**.**

**Table S10. Tukey's multiple comparisons test for the inhibition zone (mm) of *Streptococcus mutans* after treatment for 48 hours with the tested materials**

| **Tukey's multiple comparisons test** | **Mean Diff.** | **95.00% CI of diff.** | **Adjusted P Value** |
| --- | --- | --- | --- |
| PBS vs. Huge bond | -5.500 | -6.745 to -4.255 | <0.0001 |
| PBS vs. Single bond | -4.233 | -5.478 to -2.988 | <0.0001 |
| DPBS vs. G-Premio | -4.933 | -6.178 to -3.688 | <0.0001 |
| Huge bond vs. Single bond | 1.267 | 0.02182 to 2.512 | 0.0462 |
| Huge bond vs. G-Premio | 0.5667 | -0.6782 to 1.812 | 0.5021 |
| Single bond vs. G-Premio | -0.7000 | -1.945 to 0.5449 | 0.3394 |

CI: Confidence interval, DPSCs: Dental pulp- derived mesenchymal stem cells**,** DMEM: Dulbecco's Modified Eagle Medium (DMEM)**.**
